# Combining Chemotherapy Agents and Autophagy Modulators for Enhanced Breast Cancer Cell Death

**DOI:** 10.34172/apb.42733

**Published:** 2024-10-30

**Authors:** Soraya Moomivand, Mohsen Nikbakht, Ahmad Majd, Maryam Bikhof Torbati, Seyed Asadoullah Mousavi

**Affiliations:** ^1^Department of Biology, North Tehran Branch, Islamic Azad University, Tehran, Iran.; ^2^Research Institute for Oncology, Hematology and Cell Therapy Tehran University of Medical Sciences, Tehran, Iran.; ^3^Cell Therapy and Hematopoietic Stem Cell Transplantation Research Center, Tehran, Iran.; ^4^Department of Biology, Yadegar-e-Imam Khomeini (RAH) Shahre rey branch, Islamic Azad University, Tehran, Iran.

**Keywords:** Apoptosis, Arsenic trioxide, Autophagy, Breast cancer, Carboplatin, Cell lines, Cyclophosphamide

## Abstract

**Purpose:**

Autophagy, governed by genes with dual roles in cell death and survival, plays a crucial role in cancer persistence. Arsenic trioxide (ATO), carboplatin (CP), and cyclophosphamide (CY) are used to treat various cancers. ATO impedes cell proliferation and triggers apoptosis in cancer cells. CP, a platinum-based drug, damages cancer cell DNA, while CY acts as an alkylating agent, disrupting cell proliferation. This study investigates the combined effects of ATO, CP, and CY on inducing apoptosis and modulating autophagy in triple-negative breast cancer (TNBC) cell lines, BT-20 and MDA-MB-231.

**Methods:**

The cytotoxic effects of ATO, CP, and CY, alone and in combination, were evaluated using the MTT assay on BT-20 and MDA-MB-231 cells. Apoptosis and cell cycle progression were analyzed by annexin-V FITC/PI staining and flow cytometry. Gene expression of autophagy-and apoptosis-related markers, including Beclin 1, LC3, caspase 3, and BCL2, was quantified using RT-PCR. Data were analyzed using GraphPad Prism 4.0 with one-way ANOVA followed by Dunnett’s test.

**Results:**

The combination of ATO, CP, and CY significantly reduced cell viability and enhanced apoptosis, evidenced by increased caspase-3 activity and reduced BCL2 expression. Cell cycle arrest in the G1 phase was observed, alongside elevated autophagy markers Beclin 1 and LC3.

**Conclusion:**

The combination of ATO, CP, and CY induces synergistic effects in promoting apoptosis and autophagy in TNBC cell lines. These findings suggest that this combination therapy could be a promising approach to enhancing treatment efficacy in aggressive breast cancers, offering new insights into potential therapeutic strategies.

## Introduction

 Breast cancer remains the most prevalent form of cancer among women, affecting approximately 1.7 million women annually and accounting for 11.9% of the global cancer burden.^1-3^ Moreover, breast cancer constitutes 30% of all cancers in women and contributes to 15%-20% of cancer-related mortalities in the female population.^4^ Survival rates for breast cancer exhibit significant global disparities, with the estimated 5-year survival rate reaching 80% in developed countries, compared to less than 40% in developing nations.^5^ Diagnosis of breast cancer involves assessing the status of several key receptors, including the estrogen receptor (ER), progesterone receptor (PR), and human epidermal growth factor receptor 2 (HER2).^6^ Approximately 70-80% of breast cancers are hormone receptor-positive (ER, PR-positive), while 10-15% overexpress HER2, and another 10%-15% fall into the category of triple-negative breast cancers (TNBCs), which lack ER, PR, and HER2 expression.^7,8^

 Cell death is a fundamental physiological process that governs the life cycle of an organism, including growth, development, aging, and eventual death.^9^ Two primary mechanisms of programmed cell death are autophagy and apoptosis.^10,11^ Autophagy, often described as a self-degradative process, plays a dual role in cancer cells by recycling cellular components to promote survival under stress or, when overly activated, leading to autophagic cell death.^12^ Apoptosis, in contrast, is a highly regulated process that systematically eliminates damaged or malignant cells, acting as a safeguard against uncontrolled cell proliferation.^13,14^ Emerging evidence suggests that many anticancer therapies, including chemotherapy, can induce autophagy, which may contribute to both therapy resistance and tumor cell survival. As a result, targeting autophagy has been proposed as a therapeutic strategy to enhance the efficacy of cancer treatments, including in breast cancer.^15^

 Arsenic, historically known for its toxicity, has emerged as a therapeutic agent in cancer treatment, particularly through arsenic trioxide (ATO), which is widely used for its anti-cancer properties in treating acute promyelocytic leukemia (APL).^16-18^ ATO has shown promise not only in hematological malignancies but also in solid tumors due to its ability to induce apoptosis through oxidative stress and various signaling pathways.^19,20^ Alongside ATO, chemotherapeutic agents like carboplatin (CP) and cyclophosphamide (CY) are frequently employed in the management of breast cancer.^21-23^ CP, a platinum-based compound, exerts its effects by cross-linking DNA, causing significant DNA damage that leads to apoptosis in cancer cells.^2,24^ In contrast, CY functions as an alkylating agent, interfering with DNA replication and cell division, thereby inhibiting tumor growth.^25^ The combination of these agents with novel therapeutic strategies, such as autophagy modulation, offers a promising avenue for enhancing treatment efficacy in aggressive forms of breast cancer. Kuo et al have documented the efficacy of ATO as a viable intervention for refractory or relapsed APL.^26^ Therefore, it is imperative to conduct a thorough investigation into the amalgamation of ATO with other chemotherapeutic agents currently employed in clinical practice, to enhance its therapeutic effectiveness in the management of solid neoplasms in humans.

 However, the role of autophagy in breast cancer is still controversial, with its implications varying depending on the context.^27^ The aim of this study is to evaluate the combined effects of ATO, CP, and CY on the expression of autophagy-related genes such as LC3 and Beclin 1, as well as their impact on apoptosis in two TNBC cell lines: BT-20 and MDA-MB-231. This investigation will focus on determining whether varying concentrations and treatment durations of these agents enhance their cytotoxic effects, potentially offering new insights into therapeutic strategies for TNBC.

## Materials and Methods

###  Chemicals and reagents

 Fetal bovine serum (FBS), recombinant DMEM culture medium, Penicillin/streptomycin, and Trypsin-EDTA were obtained from Bio Idea Company (Tehran, Iran). Trypan Blue, chloroform, isopropanol, and DMSO were acquired from Merck (Germany). Hemocytometer slides and MTT powder were supplied by TGI (Germany) and Sigma (Germany), respectively. A manual RNA extraction solution and a cDNA synthesis kit were purchased from Genex Company (Iran). DEPC-treated water was sourced from Cinacloon (Iran). Master Mix for Real-Time PCR (2x) was provided by Amplicon Company (Denmark). All other unspecified reagents and materials were procured from Merck or Sigma.

###  Cell line and cell culture

 The breast cancer cell lines BT-20 and MDA-MB-231 were acquired from the Pasteur Institute (Tehran, Iran). Both cell lines were cultured in DMEM supplemented with 10% FBS and incubated at 37 °C in a humidified atmosphere containing 5% CO2.

###  Analysis of cell viability by MTT assay

 BT-20 and MDA-MB-231 cells were seeded into 96-well plates at a density of 5 × 10⁴ cells per well. Cells were treated with ATO and combinations of ATO with other compounds, incubated at 37 °C with 5% CO2 for 24 and 36 hours. The MTT assay was performed to assess cell proliferation, with results presented as a percentage relative to control cells treated with 0.1% DMSO. Cell viability was determined by measuring optical density (OD) at 570 nm using a plate reader. The absorbance ratio between treated and control cells was used to calculate cell viability using the following equation:


$$cellviability\left(\%\right)=\frac{\left(OD_{sample}-OD_{negetive\;control}\right)}{\left(OD_{positive\;control}-OD_{negetive\;control}\right)}\times 100$$

###  The analysis of the cell cycle

 Cells were collected and preserved in 70% ethanol. Following preservation, the cells were treated with RNAaseI (10 μL/mL) and Triton X-100 (0.1%) for 30 minutes at 37 °C. Subsequently, cells were stained with propidium iodide (PI; 10 μg /mL), and flow cytometry was employed to assess the cell cycle. Data acquisition was performed using a BD flow cytometer, and analysis was conducted with the FlowJo software. The percentage of apoptotic cells was determined by evaluating the hypodiploid G0/G1 DNA fraction.

###  Analysis of cell apoptosis by flow cytometry

 Cells were seeded into wells at a density of 15 × 10⁵ cells per well and incubated for 36 hours. During this time, cells were treated with ATO and various ATO combinations or maintained as untreated controls. Apoptosis was assessed using a staining assay with Annexin V-FITC, following the manufacturer’s instructions. The percentage of apoptotic cells was determined by analyzing Annexin V + /PI- cells using a BD flow cytometer and FlowJo software. The apoptosis analysis included the evaluation of early apoptosis (Annexin V + /PI-), late apoptosis (Annexin V + /PI + ), and necrosis (Annexin V-/PI + ).

###  RNA extraction and quantitative RT-PCR

 Total RNA was extracted using TRI pure reagent (Roche Applied Science, Germany) according to the manufacturer’s protocol. The RNA pellets were dissolved in DEPC-treated water, and RNA quality and concentration were measured using a Nanodrop ND-1000 spectrophotometer at 260 nm. cDNA was synthesized from 1-2 µg of total RNA using a cDNA synthesis kit (Takara Bio Inc., Otsu, Japan) following the manufacturer’s instructions. The concentration of cDNA was standardized, and PCR was prepared using Beclin and LC3 primers (Table 1). Real-time PCR amplification was performed using the ABI Step One Plus^TM^ system (Applied Biosystems, USA). The relative expression of selected genes was determined using the comparative Ct method. Five groups were analyzed, and gene quantification was conducted using the Rotor-Gene Q system (QIAGEN, Hilden, Germany) with the primers listed in Table 1.

**Table 1:** The primers for RT-PCR analysis

**Genes**	**Primers**	**Reference**
Beclin	F: 5’-AGCTGCCGTTATACTGTTCTG-3’	^28^
	R: 5’-ACTGCCTCCTGTGTCTTCAATCTT-3’	
LC3	F: 5’-GATGTCCGACTTATTCGAGAGC -3’	^29^
	R: 5’-TTGAGCTGTAAGCGCCTTCTA-3’	
Caspase 3	F: 5'-GACTCTGGAATATCCCTGGACAACA-3'	^30^
	R:5'- AGGTTTGCTGCATCGACATCTG-3'	
BCL2	F: 5’-CTGCACCTGACGCCCTTCACC-3’	^31^
	R: 5’-CACATGACCCCACCGAACTCAAAGA-3	
GAPDH	F: 5’-TGAACGGGAAGCTCACTGG-3’	^32^
	R: 5’-TCCACCACCCTGTTGCTGTA-3’	

###  Statistical analysis

 The data were analyzed using GraphPad Prism 4.0 (GraphPad Software, La Jolla, CA). One-way analysis of variance (ANOVA) followed by Dunnett’s test was performed to compare group means. Results were expressed as mean values with standard error (SE). Statistical significance was considered at a threshold of *P* < 0.05.

## Results

###  ATO, CP, and CY inhibit cell proliferation

 The cytotoxic effects of ATO, CP, and CY on MDA-MB-231 and BT-20 cells were assessed through the MTT assay to determine the impact of these treatments on cellular viability. The cell viability for both MDA-MB-231 and BT-20 cell lines was evaluated at two distinct time points: 24 hours and 36 hours. In the MDA-MB-231 cell line, CP administered at 1250 µM resulted in a substantial reduction in cell viability, declining from 90.67% at 24 hours to 80.29% at 36 hours. When the concentration of CP was increased to 5000 µM, significant cytotoxic effects were observed, with cell viability decreasing to 57.09% at 24 hours and further declining to 49.57% at 36 hours. Other treatments, such as CY at 200 µM and ATO at 2 µM, maintained relatively high levels of viability, with ATO at 2 µM showing minimal reduction from 89.93% at 24 hours to 88.41% at 36 hours.

**Figure 1:**
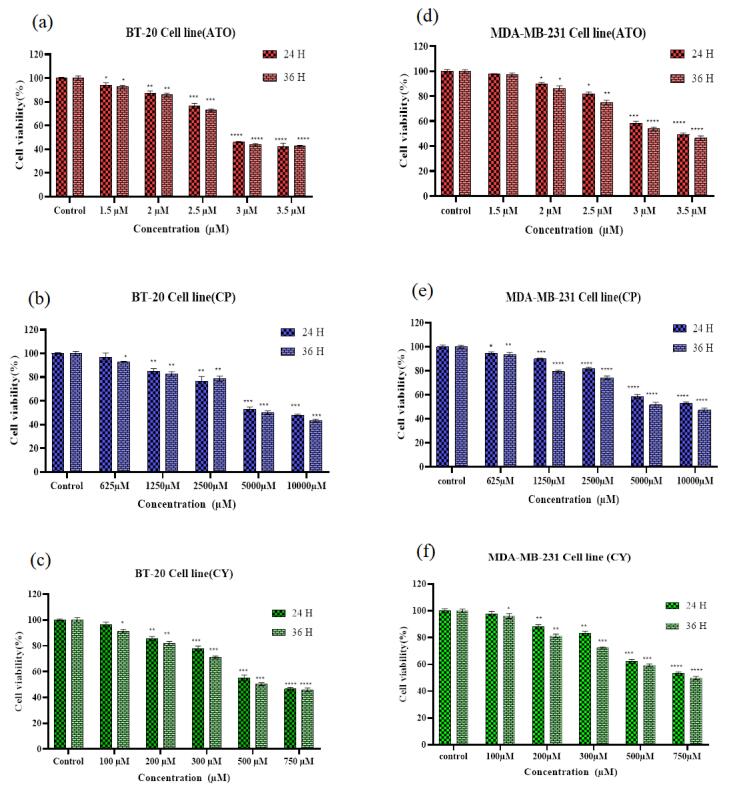
Effect of ATO, CP, and CY on cell viability in BT-20 and MDA-MB-231 cell lines, assessed using the MTT assay after 24 and 36 hours of treatment. Significant differences in cell viability were observed between the 24-hour and 36-hour treatments. (A) Effect of ATO on BT-20 cells; (B) effect of CP on BT-20 cells; (C) effect of CY on BT-20 cells; (D) effect of ATO on MDA-MB-231 cells; (E) effect of CP on MDA-MB-231 cells; and (F) effect of CY on MDA-MB-231 cells. Data are presented as mean ± standard error (SE) from three independent experiments. Statistical significance was denoted as *P < 0.05, **P < 0.01, ***P < 0.001, and ****P < 0.0001 relative to the corresponding control

**Figure 2:**
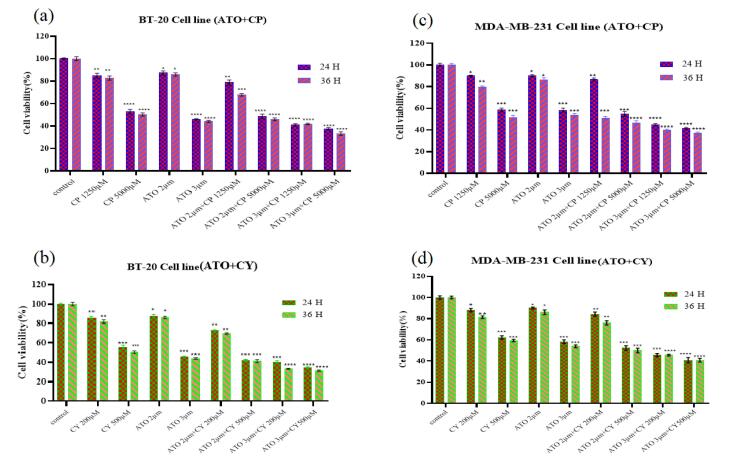
Cell viability in BT-20 and MDA-MB-231 cells was assessed using the MTT assay. The effects of ATO + CP and ATO + CY treatments were examined after 24 and 36 hours. (A) The impact of ATO + CP on BT-20 cells; (B) the impact of ATO + CY on BT-20 cells; (C) the impact of ATO + CP on MDA-MB-231 cells; (D) the impact of ATO + CY on MDA-MB-231 cells. Data are presented as mean ± SE of three independent experiments. Statistical significance was denoted as *P < 0.05, **P < 0.01, ***P < 0.001, and ****P < 0.0001 relative to the corresponding control

 Notably, combination treatments of ATO 2 µM + CP 1250 µM resulted in a pronounced reduction in cell viability, from 85.07% to 52.75%, underscoring the synergistic cytotoxic effects. Particularly, the combination of ATO 3 µM + CP 5000 µM caused a marked decrease in viability, from 40.67% to 35.94% (Figure 1 and 2). In the BT-20 cell line, similar trends were observed, with CY 500 µM leading to a significant reduction in viability, from 55.12% at 24 hours to 50.39% at 36 hours. ATO 2 µM alone maintained relatively high viability at 87.35% at 24 hours but exhibited notable declines when combined with other treatments, such as ATO 2 µM + CY 200 µM, where viability decreased from 72.83% to 69.56%. The most substantial reductions were observed in combination treatments, including ATO 3 µM + CY 200 µM, which dropped from 40.37% to 33.19%, and ATO 3 µM + CP 5000 µM, which declined from 37.62% to 33.26% (Figure 1 and 2).

###  Apoptosis assay

**Figure 3:**
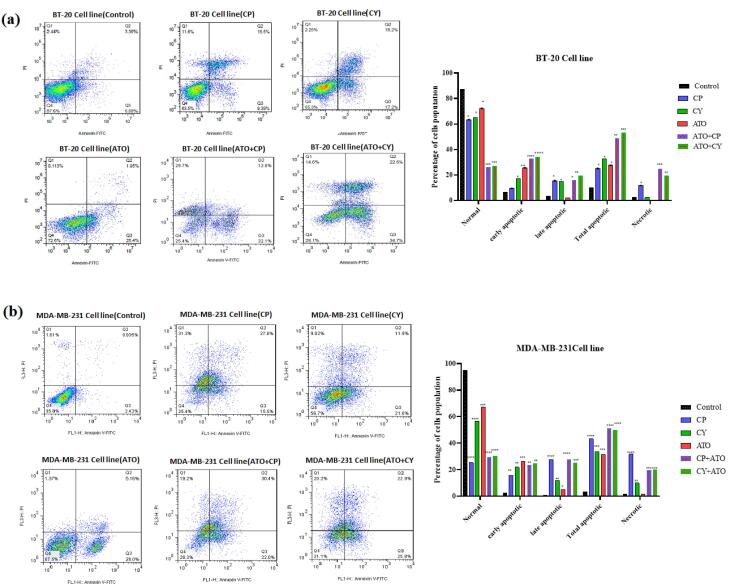
Assessment of apoptosis and necrosis using flow cytometry in (A) BT-20 cells and (B) MDA-MB-231 cells after 36 hours of treatment. Early apoptotic cells are represented in the lower right quadrant, while post-apoptotic necrotic cells are indicated in the upper right quadrant. Data are presented as mean ± SE from three independent experiments. Statistical significance was denoted as *P < 0.05, **P < 0.01, ***P < 0.001, and ****P < 0.0001 relative to the corresponding control

 To assess whether the cytotoxic effects of ATO, CP, and CY, both individually and in combination, were mediated through apoptosis or necrosis, flow cytometry analysis was conducted on BT-20 and MDA-MB-231 cell lines. Cells were treated for 36 hours and stained with Annexin-V FITC/PI, enabling differentiation between apoptotic and necrotic populations. As depicted in Figure 3a for the BT-20 cell line, 87.33% of cells in the control group were classified as viable, with a total apoptosis rate of 10.12% and necrotic cells accounting for 2.48%. In the CP treatment group, viability decreased to 63.47%, with a total apoptosis rate of 25.09% and an increase in necrotic cells to 11.77%. The CY treatment group demonstrated similar viability at 65.17%, but with a higher total apoptosis rate of 32.43% and a lower necrotic cell count of 2.35%. ATO treatment preserved a relatively higher viability at 72.33%, with a total apoptosis rate of 27.60% and minimal necrosis at 0.13%. In contrast, the combination treatment of CY and ATO resulted in a pronounced decline in viability to 28.10%, with total apoptosis reaching 57.30% and necrotic cells accounting for 14.60%. Finally, the CP + ATO combination treatment yielded the lowest viability at 25.27%, with a total apoptosis rate of 44.93% and a significant increase in necrotic cells to 29.80%.

 For MDA-MB-231 cells, in the control group, viability remained high at 95.1%, with a total apoptosis rate of only 3.28% (Figure 3b). In contrast, treatment with CP caused a substantial reduction in viability to 25.23%, alongside a total apoptosis rate of 43.17%. The CY treatment group exhibited higher viability at 56.50%, with a total apoptosis rate of 33.63%. ATO treatment maintained a relatively higher viability at 67.30%, with a total apoptosis rate of 31.30%. The combination treatment of CY and ATO further reduced viability to 31.13%, with total apoptosis increasing to 48.47%. Lastly, the CP + ATO combination treatment resulted in 28.13% viability and a total apoptosis rate of 52.40%. Based on these data, the selected doses for both cell lines were ATO at 2 μM, CP at 5000 μM, and CY at 200 μM.

###  Cell cycle assay

**Figure 4:**
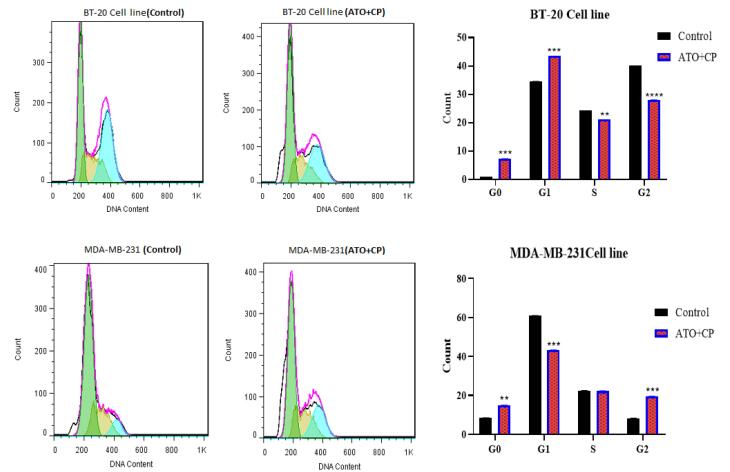
(A) Cell cycle analysis of BT-20 cells following ATO and CP combination treatment, showing alterations in cell cycle distribution. (B) Cell cycle analysis of MDA-MB-231 cells following ATO and CP combination treatment, showing a reduction in the G1 phase. Data are presented as mean ± SE of three independent experiments

 The effects of ATO and CP on cell cycle progression were analyzed via flow cytometry (Figures 4a and 4b). In the BT-20 cell line, the impact of ATO combined with CP on cell cycle distribution was assessed and compared to the control group. The results revealed significant alterations in the percentage of cells in each phase of the cell cycle following treatment. In the control group, an average of 1.025% of cells were in the G0 phase. In contrast, the ATO + CP treatment group showed a substantial increase to 7.295%, indicating a marked induction of quiescence or cell cycle arrest. In the G1 phase, the control group had an average of 34.545%, which increased to 43.525% in the ATO + CP treatment group. This increase suggests that the combination therapy may enhance the transition of cells into the G1 phase, potentially delaying their progression to the S phase. The S phase exhibited a decrease in cell population from 24.265% in the control group to 21.11% following ATO + CP treatment, suggesting that the combined treatment may inhibit DNA synthesis or disrupt cell progression through the S phase. Lastly, the G2 phase showed a decline from 40.155% in the control group to 28.05% in ATO + CP-treated cells.

 In MDA-MB-231 cells, the control group exhibited an average of 8.405% of cells in the G0 phase, which increased significantly to 14.915% following ATO + CP treatment, indicating a marked induction of quiescence or cell cycle arrest, thereby promoting dormancy in the treated cells. In the G1 phase, the control group had an average of 60.925%, which decreased significantly to 43.295% in the ATO + CP treatment group. This reduction suggests that the combination therapy may disrupt the transition of cells into the G1 phase, potentially resulting in the accumulation of cells in the G0 phase and inhibiting further progression through the cell cycle. The S phase showed a minor decrease, with the control group averaging 22.425%, and the ATO + CP treatment group at 22.265%. This slight reduction indicates that the combined treatment does not substantially inhibit DNA synthesis or significantly disrupt the overall progression of cells through the S phase, suggesting that replication processes remain largely unaffected. However, the G2 phase exhibited a notable increase from an average of 8.225% in the control group to 19.505% in ATO + CP-treated cells. This significant rise suggests that the treatment may impede the progression of cells into mitosis, causing an accumulation of cells in the G2 phase, and potentially delaying cell cycle progression, which could have important implications for the efficacy of the combination treatment.

###  A real-time assay for polymerase chain reaction

**Figure 5:**
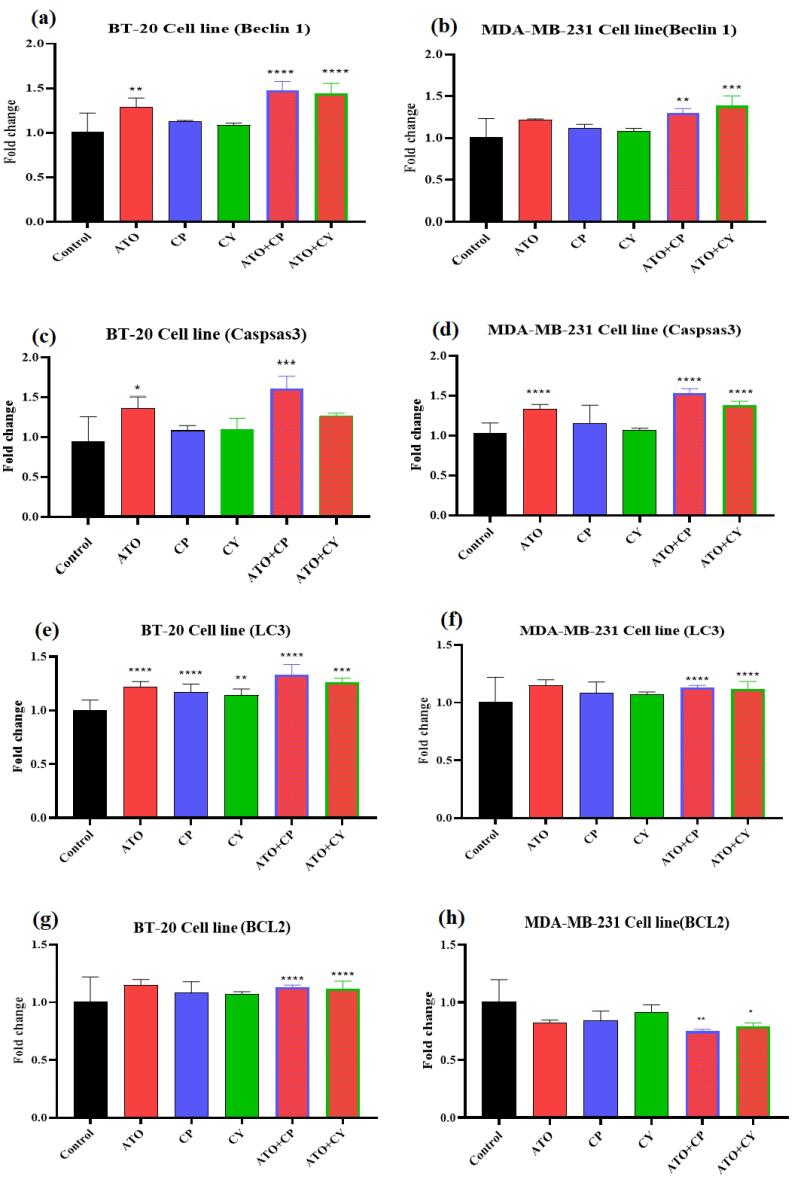
Effect of ATO, CP, CY, and their combinations on the mRNA expression levels of specific genes in BT-20 and MDA-MB-231 cells. The study assessed the effects of these treatments on the expression of (A, B) Beclin 1, (C, D) caspase 3, (E, F) LC3, and (G, H) BCL2 in BT-20 and MDA-MB-231 cells. Data are presented as mean ± SE from three independent experiments. Statistical significance was denoted as *P < 0.05, **P < 0.01, ***P < 0.001, and ****P < 0.0001 relative to the corresponding control

 To investigate the mechanisms underlying the observed effects of ATO, CP, and CY, real-time PCR was employed to assess the expression levels of Beclin 1, Caspase 3, LC3, and BCL2 genes. As shown in Figure 5a and 5b, Beclin 1 expression significantly increased by 1.34-fold and 1.21-fold in the ATO-treated group compared to the control in BT-20 and MDA-MB-231 cells, respectively. In contrast, the CP and CY groups exhibited non-significant changes. The combination of ATO with CP or CY resulted in significant increases in Beclin 1 expression in both cell lines, with the ATO + CP group exhibiting a 1.54-fold increase in BT-20 cells and a 1.44-fold increase in MDA-MB-231 cells.

Figure 5c and 5d show Caspase 3 gene expression in both cell lines. ATO treatment led to a significant increase in Caspase 3 expression by 1.43-fold in BT-20 cells, while CP and CY treatments resulted in non-significant increases of 1.13-fold and 1.26-fold, respectively. The ATO + CP combination significantly increased Caspase 3 expression by 1.65-fold, and ATO + CY resulted in a 1.33-fold increase. In MDA-MB-231 cells, ATO significantly increased Caspase 3 expression by 1.35-fold, with significant increases also observed in the ATO + CP and ATO + CY groups (1.56-fold and 1.41-fold, respectively).

 Changes in LC3 gene expression are presented in Figure 5e and 5f. BT-20 cells treated with ATO exhibited a 1.25-fold increase in LC3 expression compared to the control group, with similar increases observed in the CP and CY groups. The ATO + CP group demonstrated a 1.35-fold increase in LC3 expression, while the ATO + CY group showed a 1.25-fold increase. In MDA-MB-231 cells, LC3 expression increased slightly with ATO (1.12-fold), CP (1.15-fold), and CY (1.06-fold) treatments, though these increases were not significant. However, significant increases were observed in the ATO + CP (1.84-fold) and ATO + CY (1.55-fold) groups.

 Lastly, the changes in BCL2 gene expression across different treatment groups are shown in Figure 5g and 5h. In BT-20 cells, ATO treatment resulted in a significant 0.64-fold decrease in BCL2 expression, whereas the decreases in the CP (0.84-fold) and CY (0.85-fold) groups were not statistically significant. However, the ATO + CP and ATO + CY combinations led to significant decreases in BCL2 expression (0.64-fold and 0.76-fold, respectively). Similarly, in MDA-MB-231 cells, ATO reduced BCL2 expression by 0.82-fold, while the CP and CY groups showed non-significant decreases. Notably, the combination treatments resulted in significant decreases in BCL2 expression in MDA-MB-231 cells, with the ATO + CP group reducing expression by 0.75-fold and the ATO + CY group by 0.78-fold.

## Discussion

 Breast cancer development results from complex alterations in gene expression, occurring through genetic and epigenetic changes.^33^ TNBC, which lacks targeted hormonal therapies, poses significant challenges due to its aggressive nature. Therefore, investigating the impact of various drugs on cellular pathways is critical for identifying effective treatments.^34^ In this study, we evaluated the effects of ATO, CP, and CY on the expression of key autophagy-related genes (Beclin 1, caspase 3, LC3, and BCL2) in BT-20 and MDA-MB-231 TNBC cell lines. Autophagy plays a crucial role in maintaining cellular homeostasis by degrading damaged proteins and organelles. Disruption of autophagy has been implicated in cancer progression, invasion, and metastasis, particularly in aggressive cancers such as TNBC.^35^

 ATO, a well-known inducer of both autophagy and apoptosis, has garnered significant attention due to its ability to modulate multiple molecular pathways, including the intrinsic and extrinsic apoptotic pathways, as well as autophagic mechanisms.^36^ The present study demonstrated that ATO exhibited dose- and time-dependent cytotoxicity in both TNBC cell lines, a finding consistent with its known mechanism of action. Specifically, ATO promotes oxidative stress and mitochondrial dysfunction, leading to the activation of apoptosis through caspase-dependent pathways.^37^ Our flow cytometry analysis revealed a marked decrease in viable cell populations and an increase in both early and late apoptotic cells, further confirming the induction of apoptosis by ATO.

 Autophagy was also significantly upregulated in response to ATO treatment, as evidenced by increased expression of Beclin 1 and LC3. This finding is consistent with previous studies demonstrating that ATO induces autophagy as part of its cytotoxic mechanism, particularly in cancer cells where autophagy functions as a survival response to cellular stress.^38,39^ Chiu et al showed that ATO could synergistically induce both apoptosis and autophagy, a concept supported by our data.^40,41^ In another study, Kanzawa et al stated that ATO activates multiple intracellular signaling pathways leading to apoptosis, differentiation enhancement, or inhibition of angiogenesis.^42^ However, the role of autophagy in promoting or inhibiting cell death remains controversial, as autophagy can either protect cancer cells from chemotherapy-induced stress or lead to autophagic cell death under certain conditions.

 The present study also evaluated the combined effects of ATO with CP and CY on apoptosis and autophagy. Both CP and CY are alkylating agents that induce DNA cross-linking, leading to DNA damage and the activation of the DNA damage response (DDR) pathway. The DDR subsequently activates p53 and caspase-dependent apoptotic pathways, contributing to cell cycle arrest and apoptosis.^43^ The potential of CP as an effective anti-cancer drug, particularly for APL, was investigated by Hu et al, who found that combination therapy of high doses of ATO and CP had a synergistic effect and induced apoptosis in various solid tumor cancer cell lines; however, it is crucial to acknowledge that this treatment approach may have notable adverse effects on patients.^44^ Our results demonstrated that the combination of ATO with CP or CY significantly enhanced apoptosis, as reflected by the increased activation of caspase-3 and the downregulation of BCL2.^45^ Caspase-3 is a key executioner caspase in the intrinsic apoptotic pathway, responsible for cleaving various substrates and driving the cell toward programmed cell death.^46,47^ The activation of caspase-3 in both TNBC cell lines highlights the intrinsic apoptotic commitment induced by this drug combination. Moreover, BCL2 is an anti-apoptotic protein that plays a pivotal role in regulating mitochondrial outer membrane permeabilization (MOMP) and preventing cytochrome c release, thus inhibiting apoptosis.^48^ The combination of ATO with CP or CY appears to potentiate these effects, resulting in a robust pro-apoptotic response. Additionally, the observed reduction in BCL2 expression in both cell lines suggests that this combination therapy may sensitize TNBC cells to chemotherapy-induced DNA damage, further enhancing apoptosis.

 In conclusion, the combination of ATO with CP or CY exerts a synergistic effect on apoptosis and autophagy in TNBC cell lines, offering a promising therapeutic approach for treating aggressive breast cancers. The dual induction of autophagy and apoptosis, coupled with the downregulation of BCL2 and the activation of caspase-3, highlights the potential of this combination therapy to overcome the limitations of current treatments. Future studies should investigate the precise molecular mechanisms underlying these synergistic effects and evaluate their clinical applicability in TNBC and other difficult-to-treat cancers.

## Conclusion

 This study demonstrated that the combination of ATO, CP, and CY significantly reduced the survival and proliferation of BT-20 and MDA-MB-231 cells in a dose- and time-dependent manner. The therapy enhanced apoptosis by increasing caspase-3 activation and reducing BCL2 expression, key indicators of the intrinsic apoptotic pathway. The addition of CP and CY to ATO amplified these effects, highlighting the synergistic potential of this combination in promoting cancer cell death. The reduction in BCL2 further sensitized the cells to apoptosis, supporting the use of this combination therapy as a promising approach for breast cancer treatment.

## Competing Interests

 The authors declare that they have no conflict of interest.

## Ethical Approval

 Not applicable.
